# Chaplaincy, Spiritual Care and Moral Injury: Considerations Regarding Screening and Treatment

**DOI:** 10.3389/fpsyt.2018.00619

**Published:** 2018-12-05

**Authors:** Lindsay B. Carey, Timothy J. Hodgson

**Affiliations:** ^1^Palliative Care Unit, Department of Public Health, School of Psychology and Public Health, La Trobe University, Melbourne, VIC, Australia; ^2^Department of Religious Studies, School of Historical and Philosophical Enquiry, University of Queensland, St. Lucia, QLD, Australia

**Keywords:** moral injury, public health, chaplains, holistic care, spiritual care, religion, rehabilitation

## Abstract

Moral injury is a complex trauma related syndrome involving a correlation of biological, psychological, social, and spiritual symptoms that can have substantial impact upon health and well-being. This paper argues for a holistic bio-psycho-social-spiritual approach to moral injury, by including chaplaincy in the screening and treatment of moral injury among actively serving military members and retired veterans. As part of the moral injury treatment process, and in alignment with the World Health Organization's Spiritual Intervention Codings, a new technique is proposed, “Pastoral Narrative Disclosure” (PND), as a guide for chaplains and others trained in spiritual care to assist those suffering from moral injury.

## Introduction

Over the centuries, chaplains have been educated, commissioned, and professionally engaged to provide religious and pastoral care to military members and veterans (hereafter “personnel”) who have survived the traumatic effects of war. Across the Western World, chaplaincy as a profession remains substantive to the present day, providing ministry to a range of personnel within a variety of contexts (1). Moving beyond the traditional terminology associated with religious or pastoral care, the revision of the WHO-ICD-10 “Pastoral Intervention Codings” (2, 3) subsequently led the World Health Organization (2) reaffirming the various chaplaincy services into five categories of “spiritual intervention” codings (colloquially abbreviated as the “WHO-SPICs”; refer Table 1).

**Table 1:** WHO spiritual care intervention codings (“WHO-SPICs”).

**Summary Table (WHO-ICD-10-AM/ACHI/ACS, July,2017)^(a)^**		
**IBN^(b)^**	**Procedure code**	**Spiritual care intervention descriptor^(c)^**
**1824**	**96186-00**	**Spiritual assessment**—Initial and subsequent assessment of wellbeing issues, needs and resources of a client. This intervention can often lead to other interventions. Includes: informal explanatory dialogue to screen for immediate spiritual needs including religious and pastoral issues and/or the use of a formal instrument or assessment tool.
**1869**	**96086-00**	**Spiritual counseling, guidance, or education**—An expression of spiritual care that includes a facilitative in-depth review of a person's life journey, personal or familial counsel, ethical consultation, mental health, life care, and guidance in matters of beliefs, traditions, values and practices.
**1915**	**96187-00**	**Spiritual support**–The provision of a ministry of presence and emotional support to individuals or groups. Includes: companioning of person(s) confronted with profound human issues of death, dying, loss, meaning, and aloneness, emotional support and advocacy, enabling conversations to nurture spiritual wellbeing and healing, establishing relationships and hearing the person(s) narrative.
**1915**	**96240-00**	**Spiritual ritual**—All ritual activities both formal and informal. Includes: anointing, blessing and naming, dedications, funerals, meditation, memorial services, private prayer, and devotion, public and private worship, rites, sacraments, seasonal and occasional services, weddings, and relationship ceremonies.
**1916**	**95550-12**	**Allied health intervention—spiritual care** (Generalized Intervention) [Includes: any spiritual care intervention undertaken that is not specified or not else-where classified].

(a) *Summary Table developed from: (i) Australian Consortium for Classification Development (ACCD) (4), p. 235 and (ii) Australian Consortium for Classification Development (ACCD) (5). Spiritual: (i) assessment, p. 262; (ii) counseling, guidance and education, p. 272; (iii) support, p. 291; (iv) ritual, p. 291; (v) allied health intervention—generalized intervention (listing only), p. 291*.

(b) IBN, Intervention Block Number;

(c) *Tabular listings previously classifying “pastoral care” or “religious” interventions are now indexed under the above “spiritual care” procedural codes*.

The WHO-SPICs are useful for chaplains and other spiritual carers to formally notate their spiritual screening and treatment interventions used to assist the health and well-being of their clients. It is arguable however, that the naming of the WHO-SPICs and associated interventions were only possible given a common understanding and consensus definition of the term “spirituality”: “Spirituality is that aspect of humanity which refers to the way individuals seek and express meaning and purpose and the way they experience their connectedness, to God, to self, to others, to nature, and to the significant or sacred” [(6), based on (7)]. While not all academics and health care practitioners agree with this definition, nevertheless it has (or similar variations) become increasingly utilized across medical, nursing and allied health professions (including chaplaincy) by providing a common understanding internationally of what “spirituality” means.

While the term “spirituality” seems to have reached a definable consensus, this is not the case with moral injury. Defining “moral injury” has proven a difficult task. Lancaster et al. [(8), p. 15] note that there have been at least 18 different conceptualizations regarding moral injury since the original concept by Shay, (9, 10). It is not however, the intent of this paper to revisit all the various definitions which have previously been reviewed (11). Essentially, what is important for chaplains, and the chaplaincy profession, is that a definition of moral injury be holistic and multi-disciplinary based upon a twenty-first century bio-psycho-social-spiritual paradigm (12, 13). The definition of moral injury, for the purposes of this paper, is an amalgamation of the reviews of both Jinkerson (14) and Hodgson and Carey (11), namely:

“Moral injury is a trauma related syndrome caused by the physical, psychological, social and spiritual impact of grievous moral transgressions, or violations, of an individual's deeply-held moral beliefs and/or ethical standards due to: (i) an individual perpetrating, failing to prevent, bearing witness to, or learning about inhumane acts which result in the pain, suffering or death of others, and which fundamentally challenges the moral integrity of an individual, organization or community, and/or (ii) the subsequent experience and feelings of utter betrayal of what is right caused by trusted individuals who hold legitimate authority.

The violation of deeply-held moral beliefs and ethical standards—irrespective of the actual context of trauma—can lead to considerable moral dissonance, which if unresolved, leads to the development of *core* and *secondary* symptoms that often occur concurrently. The core symptoms commonly identifiable are: (a) shame, (b) guilt, (c) a loss of trust in self, others, and/or transcendental/ultimate beings, and (d) spiritual/existential conflict including an ontological loss of meaning in life. These core symptomatic features, influence the development of secondary indicators such as (a) depression, (b) anxiety, (c) anger, (d) re-experiencing the moral conflict, (e) social problems (e.g., social alienation) and (f) relationship issues (e.g., collegial, spousal, family), and ultimately (g) self-harm (i.e., self-sabotage, substance abuse, suicidal ideation and death)”.

### Moral Injury and Post-traumatic Stress Disorder

Some attempts have been made to distinguish moral injury from post-traumatic stress disorder (PTSD). Essentially however, this can be a difficult task, because there exists a degree of overlapping issues. Trauma-related conditions arise from exposure to a trauma event, which in the case of PTSD, results in the altered belief about safety (e.g., “the world is a dangerous place in which I live in fear”), as distinct from moral injury which is multifaceted and involves a person's altered beliefs about meaning, purpose, faith or spirituality (e.g., “there is no hope”). That is, PTSD is essentially a fear based anxiety disorder caused “after a person is exposed to actual or threatened death, serious injury or sexual violation” [(15), p. 43], whereas moral injury is a “broad bio-psycho-social-spiritual sequeala,” which we believe can exist as an independent syndrome and can indicate a “risk factor for impaired life functioning and development, or worsen several psychiatric disorders” [(13), p. 2446].

While some suggest a non-syndromal approach to classifying moral injury [e.g., (16), p. 392], nevertheless we argue, that for appropriate screening and treatment, moral injury should be understood as an “eclectic of injuries”—involving “biological/physiological injury,” “psychological/emotional injury,” “social/familial injury,” and “spiritual/religious injury”—each having a variety of symptoms, some of which are in-common, while other symptoms are unique to a particular “injury” (Table 2). That is to say, in order to screen for moral injury and understand the functional impact of moral injury upon the individual—so as to subsequently engage appropriate treatment—it is essential to identify the key symptoms. What is also essential to understand, for the screening and holistic treating of moral injury, is that moral injury is not simply physiological, nor solely psychological, or just social/cultural, nor is it purely based upon spiritual injury or moral pain, but rather moral injury is a four dimensional bio-psycho-social-spiritual infliction with a variety of interwoven symptoms.

**Table 2:** Moral injury—Bio-psycho-social-spiritual symptoms^(a)^.

**Biological/** **physical injury**	**Psychological/** **emotional injury**	**Social/** **familial injury**	**Spiritual injury**
Insomnia “Startle-reflex” Alcohol abuse Drug addiction Loss of memory Self-sabotage / Self-harm Suicide	Anger & Betrayal Shame, Guilt, Sorrow Loss of trust in self Loss of trust in others Fear and Anxiety Re-experiencing the moral conflict/Flashbacks Nightmares Gambling addition Sexual/Porn Addiction Self-deprecation Loss of self-worth Depression Suicidal ideation	Spousal/Partner Disconnection Child-Parent Disconnection Family Disconnection Collegial Disconnection Occupational dysfunction Professional Disconnection Legal and disciplinary issues Community/Cultural Disconnection Social Alienation	Anger & Betrayal Shame, Guilt, Sorrow Loss of trust in self Loss of trust in others Loss of faith/ belief Moral pain /dissonance Questioning morality Self-condemnation Spiritual/existential crisis Loss of purpose in life Fatalism Loss of caring Ontological loss of meaning. Feeling “haunted”

Source:

(a) *Hodgson and Carey (17)*.

## Moral Injury Screening and Chaplaincy

As indicated by the definition (noted earlier), moral injury should be regarded as a complex phenomenon involving physiological, psychological, social, and spiritual issues, so perhaps it should not be surprising that as yet there still remains no single validated instrument ideally recommended for chaplains that can be readily utilized for the screening of moral injury and spirituality. There is however, literature which supports the involvement of chaplains undertaking screening evaluations for moral injury prior to or during their intervention of spiritual counseling. Indeed some literature indicates that chaplains can be an important and initial “port-of-call” for screening veterans who may potentially be suffering a moral injury.

For example, Nieuwsma et al.'s research (18), surveying US Veteran Affairs' chaplains (*n* = 440) and US Department of Defense chaplains (*n* = 1,723) indicated that 14% of DoD chaplains and approximately 45% US Veteran Affairs, had “*frequently”* met with and provided support to personnel suffering from moral injury. While the majority of DoD chaplains (59.5%) acknowledged being only involved “sometimes” with military personnel whom they believed were suffering a moral injury, this nevertheless indicates that a substantial number of military chaplains were connecting (even if only “sometimes”) with personnel potentially showing symptoms and/or signs of moral injury. It seems logical that both military chaplains and veteran affair chaplains should be considered valuable front-line “reconnaissance” for identifying and helping to formally screen those with potential symptoms and signs of a moral injury.

Indeed, medical specialists involved in veteran care, such as Kopacz et al. (19), suggest that chaplains should at least utilize spiritual screening scales that are currently available [e.g., “Spiritual Distress Scale”—SDS; (20)], so that chaplains can empirically identify those personnel who are “at risk” [e.g., suicide ideation behavior; (21)], and thus “allow chaplains to be more responsive” to the spiritual and pastoral needs of those for whom they are required to provide care (19). As reviewed by Drummond and Carey (22); Carey et al. [(23); p. 12], there are in fact numerous evaluations that can be used by chaplains for the screening and assessment of religious and spiritual issues affecting the health and well-being of their clients. Amidst these instruments, there are a number of tools that chaplains could utilize which focus specifically upon factors/symptoms relating to moral injury—examples of these instruments are provided at Table 3.

**Table 3:** Examples of moral injury spiritual screening tools accessible/utilized by chaplains^(a)^.

**Instrument**	**Key focus**		**Specialty**
Spiritual injury scale/index (24)	Guilt Anger or resentment Grief or sadness Lack of meaning or purpose Despair or hopelessness	Feeling that God/life abandoned Religious doubt or disbelief Fear of death	Mental Health Spiritual Injury Moral Injury
Impact of Event Scale—Revised (IES-R) (25)	Traumatic Events Intrusion into life	Hyper-arousal Avoidance	Mental health Military & veterans PTSD/Moral injury Health & emergency service personnel
Moral Injury Events Scale (MIES) (26)	Betrayal Morality	Immorality Ethics	Mental health Military & veterans PTSD/Moral injury
Spiritual Distress Scale (19)^(a)^	Guilt Sadness/grief Resentment	Anger/ Despair/hopelessness	Mental Health Military & Veterans PTSD/Moral Injury Suicide
Moral Injury Questionnaire—Military (MIQM) (27)	Betrayal Guilt Retribution Humanization	Violence Destruction Death	Mental Health Military & Veterans PTSD/Moral Injury
Modified Military Moral Injury Questionnaire (M3IQ) (11)^(b)^	Immoral acts (witnessed and/or perpetrated) Death/injury (civilians, military, enemy) Betrayal (self & others)	Ethical dilemmas (decision-making, humanization) Disproportional violence/retribution Grief, shame and unresolved issues	Moral injury Existential/spiritual Ethics/morality Military & veterans
Moral Injury Symptoms Scale—Military (MISS-M) (28)	Betrayal Guilt Shame Moral concerns Religious struggles	Trust Meaning/purpose Forgiveness Self-condemnation	Mental Health PTSD Moral Injury Military & Veterans

(a) Instruments presented in chronological order ^(a)^(29) developed from (24) Spiritual Injury Scale;

(b) *M3IQ: Based on the MIES (26) and the MIQM (27)*.

While some chaplains may be open to undertaking moral injury screenings as part of their pastoral ministry/spiritual care, others may need educating about the benefits of undertaking screenings as part of a recognized WHO-SPIC spiritual assessment intervention (refer Table 1). Of additional value would be chaplains becoming involved in, or even initiating, the development of instruments to assist with the screening and treatment of moral injury. For example, to identify those “at risk” and to enable chaplains to be more responsive to those personnel potentially suffering moral injury, the first exploratory research undertaken within the Australian Defence Force (ADF) was initiated, not by psychiatrists, not by psychologists, nor social workers, but implemented and supported by chaplains who were genuinely concerned about the well-being of war veterans (17).

A 100 item “Modified-Military-Moral-Injury-Questionnaire” (M3IQ) was initiated by chaplains to implement a preliminary screening to assess whether or not any Australian military personnel had experienced a potentially morally injurious event while on deployment. Whereas a number of previous studies regarding moral injury focused upon military personnel who were already diagnosed with post-traumatic stress disorder, the uniqueness of the M3IQ was its focus upon those personnel who had ***not*** been diagnosed with PTSD, yet the majority still evidenced symptoms of a “moral injury” post-deployment (17). While the analysis of the M3IQ research results are currently being undertaken and will be of interest to medical, nursing, and allied health professionals alike, nevertheless the involvement of chaplains at the initial screening level helps to ensure a truly holistic bio-psycho-social-spiritual approach as part of a continuum of care—from screening to treatment—which includes the involvement of chaplains as part of a multidisciplinary approach toward moral injury rehabilitation. It is important to note however, that while chaplains may be involved in the screening for moral injury, this does **not** mean that chaplains would solely be responsible for diagnosis or subsequent treatment. Effective intervention needs both the appropriate mental health professional and the chaplain working in collaboration. Indeed a chaplain failing to make reference to mental health professionals could potentially cause harm, as could mental health professionals by not referring appropriately to chaplains. Wortmann et al. [(30), p. 258] summarize recommendations for when clinicians should consult with and/or refer to chaplains/clergy regarding the treatment of moral injury. These include when personnel show symptoms and/or signs of (i) persistent guilt or shame after perpetration, (ii) anger and/or mistrust after betrayal, (iii) intense, chronic negative self-evaluation linked to religious/spiritual beliefs and (iv) alienation from the community. While these are good recommendations for referral/consulting with chaplains/clergy, we would add (given that chaplains are often regularly involved in the lives of personnel) that clinicians should also consult/refer to chaplains/clergy (v) given personnel performance work place issues and (vi) familial issues.

## Moral Injury Treatment and Chaplaincy

While there is considerable international variation with regard to the nomenclature for classifying a “chaplain,” nevertheless when discussing issues in relation to health care treatment, it is important to distinguish for the purposes of this article, between “community clergy” (e.g., parish clergy, assistant and/or volunteer spiritual carers, etc.) and a “chaplain” (e.g., certified/clinically trained health care chaplains, military chaplains, veteran affairs chaplains, etc.). The majority of chaplains have completed additional training (specific to their industry/sector) beyond the standard religious, theological, and/or parish education/experience. While there is evidence of community clergy receiving and/or providing moral injury training in collaboration with mental health and other specialists (31, 32), nevertheless most of the literature regarding the beneficial and influential role of chaplains, has been within the health care sector (33, 34)—even more so with respect to mental health care (23, 35, 36).

Some of the health care literature has considered the specialist role of military and veteran affair chaplains. Hale (37), for example, surveying US Navy personnel, found that the majority (*n* = 213/250: 85.2%) either “agreed” or “strongly agreed” that their “chaplain/pastoral care service was best qualified to treat their spiritual/moral injury”. Nieuwsma et al.'s research [(18); noted earlier] indicated that the majority of both DoD (62%) and VA chaplains (66.4%) believed that their chaplaincy training made them “very prepared” to provide pastoral support for those experiencing moral injury [(18), p. 132]. Kopacz et al. (38) was one of the first to argue that “those affected by [moral injury] may benefit from more than just conventional mental health services'. They noted four distinct advantages of pastoral care that may be helpful by: (i) resolving some of the dynamic issues underpinning moral injury (such as forgiveness and guilt), (ii) assisting military and veteran personnel who have embraced a religious/spiritual identity with coping/resilience, (iii) providing familiarity, given that personnel within the military are (via the role of the chaplain) accustomed to such a supportive role which, (iv) does *not* encompass an imposition of values or beliefs but is sensitive to the individuals own spirituality and sense of meaning and purpose [(38), p. 31].

In responding to the condition of moral injury, many chaplains have in the past, as part of their pastoral/spiritual care ministry to those of religious beliefs, used a confessional process (of one kind or another), traditionally called the Sacrament of Penance *(or “Sacrament of Reconciliation”*, seen as a sacrament of healing) which is considered “*sacrosanct*” (i.e., too important or valuable to be altered) and encouraged personnel to name their experience of moral injury and seek forgiveness. Such a ritual process may still have merit today, as it can complement other therapeutic processes of various health carer practitioners.

Traditionally, the Christian religious confessional process consisted of: (i) contrition, (ii) confession, (iii) penance, and (iv) absolution [(39), p. 165–166]. In comparison Litz et al. have carefully crafted “Adaptive Disclosure Therapy” (ADT) (40, 41), which is an adapted or “secularized form of the ‘sacrament of penance’ modified with the critical exclusion of the priest” [(42), p. 1]. However, given the religious, spiritual, existential and ethical issues associated with moral injury, the role of the clergy/chaplain may be critical, and thus there is a need to reconsider traditional practices, and utilize new terms that embrace the spirituality of all personnel—whether they be of a religious faith or none.

## Pastoral Narrative Disclosure

The sacrament of penance recognized and acknowledged the moral pain of returned military personnel which encouraged them to return to families and the community—absolved, forgiven, and cleansed. Such sacramental practices applied today however, would be foreign to the social and cultural experiences of many present day personnel and possibly seem meaningless or even inappropriate to those of non-Christian religions. Nevertheless, given that moral injury seems to transcend religious/spiritual perspectives (43), and that chaplains are quite apt at providing cross-cultural ministry to those of no faith and those of any faith (44, 45), chaplains could uniquely adapt aspects of these traditional practices to help present day personnel address their moral injury.

While there exists some excellent therapy techniques that could be used to model a moral injury intervention [e.g., Religiously Integrated Cognitive Behavior Therapy; RCBT (46)], nevertheless similar to Litz et al's ADT (41), we have developed a “*Pastoral Narrative Disclosure*” (PND) intervention specifically for use by chaplains. PND is based on a liturgical confessional model empirically evaluated by Joób and Kettunen (39) and includes the work of Verkamp (47) regarding the “moral treatment of returning warriors”. The three locutions of “pastoral,” “narrative” and “disclosure” are deliberately used. Firstly, “pastoral” which embraces the individual holistically, secondly “narrative,” which embraces the individuals story as part of their being, and finally “disclosure,” which is a more modern term for confession.

PND is fundamentally a revised confessional model that has been in place for centuries and is largely still utilized (in one form or another) by many clergy/chaplains. PND is not a theological discourse but a health care intervention which seeks to provide a model for the effective application of spiritual and pastoral care. We have categorized the PND model into eight stages to gain feedback from the wider professional community and to make the PND process statistically assessable and testable for validity so as to ensure PND credibility. The eight “R” phases of PND (summarized at Table 4) identify the chaplain's role in order to help personnel explore the experience of moral injury, consider guilt and shame, seek forgiveness, and reconnect with themselves, their family and their community. Consisting of eight (proposed) 60–90 min sessions, each of the phases presented are sequential in order, nevertheless it may be appropriate at times to return to previous phases depending on the progress and issues raised by personnel.

**Table 4:** Pastoral narrative disclosure (PND)^(a)^—Eight stage summary for the spiritual counseling and education^(b)^ of personnel experiencing moral injury.

**Stage**	**Summary explanation**
1. Rapport	Developing rapport/trust between personnel/service member and chaplain, who ensures (caveats permitting) absolute confidentiality.
2. Reflection	Personnel/service member provides an account either oral, written or by other medium, reflecting upon operational life journey and their morally injurious experience.
3. Review	Indepth review of personnel/service member's reflection regarding their morally injurious experience by examination of conscience—considering past thoughts, words, actions, and omissions, particularly with regard to self-accusation/s.
4. Reconstruction	Reconstruct the moral/ethical issue relating to the event and address feelings of grief, guilt, shame, anger, betrayal, trust, and forgiveness.
5. Restoration	Restoration is sought regarding grievances, which if possible, are heard by the perpetrator or organizational representative.
6. Ritual	Rituals, either formal or informal, secular or religious rites, expressing regret, naming mistakes, change of heart, and seeking self-forgiveness and/or forgiveness from a significant or sacred source.
7. Renewal	Engaging in renewal by personnel/service member making amends and doing activities that are meaningful/purposeful in life by relinking with family, friends, workplace, community, the sacred/divine/God.
8. Reconnection	Reconnection involves personnel/service member engaging support and resources to reconsider or implement future values, career plans and personal goals relevant for themselves and significant others so as to develop resilience and sustain themselves long term.

(a) *Source: Carey and Hodgson, this paper*;

(b) *WHO-ICD-10-AM Spiritual Intervention Coding “Spiritual Counseling and Education” (refer Table 1)*.

### Rapport

Military chaplains are uniquely appointed within their respective defense force to provide confidential counseling services sailors, soldiers and airmen/airwomen, ensuring personnel can wholeheartedly trust their chaplains in what they discuss with them without fear of reporting, reprimand or reprisal (48). For example, in the United States military, “Rule 503: Communication to Clergy,” states “a communication is ‘confidential’ if made to a clergyman in the clergyman's capacity as a spiritual adviser or to a clergyman's assistant in the assistant's official capacity and is not intended to be disclosed to third persons” [(49), p. III-24]. Thus, US military chaplains cannot be ordered by the chain of command to write assessments or reveal any information about particular personnel. This is also the case, for example, within other defense forces such as the UK Armed Forces, Australian Defence Force, and the New Zealand Defence Force, whose chaplains provide “absolute confidentiality” (with certain caveats) for all personnel [(50), p. 5; (51, 52)]. Overall, given such a level of privacy held by the chaplain, combined with their military experience, helps to increase trust, which has been argued to be particularly important so as to lead to more favorable health outcomes (53).

### Reflection

Due to the rapport developed, personnel returning from deployment, may share or disclose with a military chaplain their experience of operations (warlike or non-warlike). Needing to “offload” a narrative that is immersed with guilt, shame or anger, is an important cathartic step, as a moral injury can potentially define and consume their entire being. Reflective spiritual care “begins with lamenting the shared anguish of moral injury” [(45), p. 1] and involves personnel providing either oral, written or other type of medium (e.g., pictures, video clip) about their morally injurious experience.

Particular themes (e.g., betrayal or perpetration) and/or symptoms (e.g., anger, guilt or shame) would be identified by the chaplain which then permits a review process. Recognizing the pivotal role that narrative has in understanding personnel's operational experience, such praxis provides a conjuncture between their “war story,” their “previous stories,” “community stories,” and “faith stories”. It is important that personnel “own their story” by embracing all the anguish and hurt which it may cause, coming to an acceptance that progresses toward their cleansing and wholeness. Overall PND involves not just stories told, but stories of “one's being” or “one's self” and about the meaning of their life. Given the powerful effect of the reflective process, it is of course important that chaplains have access to additional resources and health care personnel. This is to ensure a safety barrier so as to effectively address the negative emotions from some personnel given the indepth re-telling of their morally injurious event/s. Potentially there is a real risk of producing further trauma by the re-telling of traumatic events. However the process of reflection and the essential importance of the chaplain's role with regard to moral injury, predominantly concerns the person's faith, beliefs, or framework for meaning, rather than purely traumatic events. Bussing et al's research among military personal, has indicated that “…the process of life reflection and subsequent intention to solve conflicting situations and experiences, can be considered as a process to cope with one's own failures, guilt, and mistakes” (54). This reflective stage provides the opportunity to consider the moral and spiritual impact of failures, guilt and sense of betrayal rather than just focusing on the trauma.

### Review

Following a person's reflection, an in-depth critical self-review of their operational life experience should be undertaken. It would involve an examination of conscience by personnel, facilitated by the chaplain, to critique their or other's conduct on operations. Any reflective self-accusation would be noted by the chaplain who would identify particular symptoms and themes. A further concept for consideration is collective guilt, whereby personnel associate their examination of conscience with their nation state's participation in an event and its culpability. Additionally, personnel can associate their examination of conscience, whereby their behavior could be considered against particular ethical principles or sacred texts (if appropriate) that provide precise relevance and personal meaning [(47), p. 96, (55), p. 3, (56), p. 37].

After concluding an examination of conscience, personnel may identify that they have done nothing wrong and their feelings of anger, shame, or guilt are largely undeserved. With the help of chaplains, social workers or psychologists, personnel will need to be taught how to accept such feelings. It is also quite possible that personnel, after examining their conscience, may realize that their deep sacred beliefs have been violated by themselves. Even if their actions are within the rules of engagement or the laws of armed conflict, they may still struggle with their conscience [(47), p. 98]. This leads to the need for moral reconstruction.

### Reconstruction

Reconstruction, with regard to moral injury, is the rebuilding of a person's belief system which has been fractured by their morally injurious experience. Most military chaplains have a solid academic foundation as a result of their theological training in ontology, moral theology, ethics and reflective praxis. Given their additional specialist military training and active service experience, chaplains can help personnel to explore their moral conscience and why a morally injurious event has affected them. The chaplain's role requires that he/she be conscious of the needs of the whole person, including their physical, psychological, social, and spiritual issues, which can emerge as a result of a morally injurious event. The military chaplain can help personnel explore the ethics and morality behind the event and the person's involvement. As part of the reconstruction phase, the chaplain can address with personnel the issues of grief, guilt, shame and anger, plus rebuild the values of trust, and forgiveness (45).

### Restoration

To respond to moral injury issues that involve betrayal, a restorative process may be necessary if possible. Betrayal is considered a fundamental “assault on human dignity and brings with it powerful disappointment and discouragement” (57). The issue of betrayal, be it by others or self-betrayal, requires a restoration to allow the person to have their grievance heard either by those directly involved in the incident (e.g., perpetrator/chain of command) or a senior defense member representing the defense institution/service. Such a process enables a reciprocal conversation of truth and understanding to take place, whereby the person may confirm their experience or gain further information to better understand the wider context. Most importantly restoration enables the possibility for the relationship between the person and the institution/service to be restored, as “repair is not only material loss or damage, but a state of relationship that has been shaken, broken, distorted, or fouled” [(58), p. 209]. This phase will preferably involve a verbal acknowledgment or apology from the perpetrator or from a senior defense representative, however if it is not possible for a face-to-face meeting, a written document may suffice.

### Ritual

Even though not all personnel are religious, many have had a spiritual upbringing or influence, where religion has played a fundamental or at least a serendipitous role in the development of their moral worldview. Thus, whether consciously or unconsciously, many personnel would associate their morality based on one or more narratives of a traditional faith structure (e.g., Buddhist, Christian, Hindu, Islam, Judaism, etc) or an idiosyncratic combination of religious beliefs. Traditionally throughout the ages, military members often sought a priest/cleric/chaplain/rabbi (or equivalent) to conduct a ritual such as a prayer of confession (or equivalent disclosure). By clearing their moral conscience, a first step was taken toward reversing their potential debilitation. Whether the type of moral injury is betrayal or perpetration, particular types of rituals according to different faith/religious perspectives, can be utilized to help personnel treat and cleanse their moral injury. Following a “confession,” personnel may seek absolution from a cleric/chaplain. This may include not only a petition for forgiveness from a divine being/God but also forgiveness from others.

An additional challenge for personnel is also the struggle to forgive themselves and even to forgive God (or other divine entity) for failing to intervene [(47), p. 103–104]. As part of the prayers of forgiveness, other rituals can be utilized such as (in the Christian tradition), the chaplain/priest sharing the eucharist, anointing personnel with oil and/or conducting prayers of restoration. As noted by Hughes (59), prayer can help personnel to “feel empowered to heal and/or to be reconciled with the divine, the faith community, or significant relationships in his or her life” (p. 58). As a matter of authenticity, genuineness and integrity, and irrespective of the religious/spiritual tradition, this ritual role should be undertaken by an authorized serving religious practitioner (e.g., cleric/chaplain).

### Renewal

Renewal, colloquially expressed, means commencing life “a new” with a “clean slate”. Using a “ritual of penance” (i.e., “making amends”) or similar, is a method to help personnel engage in doing new activities that are life enriching. Penance provides a means or “route away from self-destructive patterns and toward life-affirming strategies” [(60), p. 79].

One of the consequences of a moral injury is that it causes a rupture in relationships between working personnel, family members, community or religious/spiritual affiliations, and thus potentially leading to alienation. This relational-rupture may be due to a perception of self-agency (e.g., “I could and should have done something”) or through a loss of faith in God (e.g., “Why did God allow this?”), or perhaps a loss of trust in the community or a damaged anthropology (e.g., “All people are evil”). Regardless, the impact of this rupture evidences the need for utilizing PND to encourage healing and renewal.

Renewal involves ensuring personnel achieve effective working and supportive relationships in: (i) the workplace; (ii) with their spouse/partner, children and extended family; and (iii) in the wider text such as faith communities, thus encouraging opportunities for communication and developing relationships (61, 62). For example personnel may spend preplanned weekends away with their family to encourage positive communication opportunities, or engage in community activities together.

### Reconnection

Reconnection involves personnel considering and engaging support and resources to reconsider their current and future values, plans and goals relevant for themselves and their significant others. This may involve revisiting and reconsidering previous work of the initial PND process (e.g., reflection and review) so as to discern any unresolved issues and/or plan strategies to move forward. To enable future stability, personnel are encouraged by the chaplain to connect with wider support and resources. With the approval of the person concerned, additional support from general practitioners, nurses, psychologists, social workers, community clergy, chaplains, and other allied health professionals, will assist the member to maintain their progress and develop their resilience.

## Conclusion and Recommendations

No doubt some clinicians, for the purpose of seeking to maintain and extend their professional boundaries, will believe their unilateral conceptual frameworks of addressing moral injury are exclusively correct—they will prefer frameworks that are not truly holistic, failing to endorse a multidisciplinary approach. Some will attempt to exclude or minimize the role of chaplains or clergy down to occasional referrals or argue that the chaplaincy role can be accomplished by non-religious personnel or even replace the chaplain with an empty chair! Given the complexity of moral injury however, it is important for “medical, nursing and allied health personnel to work alongside chaplains to assist with moral injury rehabilitation. This will ensure that healthcare departments/facilities do not ‘adopt a stance that excludes the significance of spirituality,’ nor minimizes spiritual interventions due to professional demarcation at the expense of client wellbeing” [(63), p. 245; (64), p. 40]. Most certainly PND itself is intended to be used, not in isolation, but as part of a multidisciplinary approach.

This is particularly important if personnel have post-traumatic stress disorder combined with moral injury, thus requiring the effective intervention of both the appropriate health care professional and the Chaplain in addressing dual conditions.

Another advantage of PND, is that while some religious traditions (and individual chaplains or personnel), might be more comfortable with a traditional “confessional” model, an adapted application such as PND, can be utilized across different traditions and faith paradigms, making it quite versatile. Further, as it is unlikely that health care professionals will become more resourceful by undertaking theological education, it is recommended therefore that chaplains “have to be proficient in at least three languages to work alongside medical, nursing and other allied health practitioners; namely, *clinical* language, *cultural* language and the language of the *personal/spiritual*. Holding these three languages in creative tension is vital for genuine person-centered and holistic care” [(63), p. 246]. This will allow chaplains to be multi-literate so as to facilitate collaborative teamwork.

As noted earlier however, some chaplains may be reluctant to utilize any moral injury screenings or PND. A duty of care however, may warrant the need for chaplains to be educated about the benefits of using available screening instruments—not for the purposes of reporting, *per se* (for this would be a breach of confidentiality), but rather to assist chaplains to more empirically assess the narratives of their clients and ultimately assist their client's needs. In order, however, for chaplains to progress their clients beyond screenings and assessment interventions, there is also a need for chaplains to develop and utilize a systematic method of providing spiritual counseling and education, plus incorporate ritual activities to address moral injury. The proposed PND technique is one way that chaplains may be able to encourage and achieve the appropriate spiritual care interventions, that will ultimately provide personnel with the beneficent support which they need to address their moral injury.

## Ethics Statement

This research was developed with the support of the University of Queensland Human Research Ethics Committee (Brisbane Australia), the Australian Defence Force Human Research Ethics Committee (ADHREC) and the Palliative Care Unit, La Trobe University (Melbourne, Australia) in compliance with the (Australian) National Statement on Ethical Conduct in Human Research, Canberra: National Health and Medical Research Council.

## Author Contributions

All authors listed have made a substantial, direct and intellectual contribution to the work, and approved it for publication. The PND technique forms part of the Ph.D. research of the co-author TH.

### Conflict of Interest Statement

The authors declare that the research was conducted in the absence of any commercial or financial relationships that could be construed as a potential conflict of interest.
